# Preliminary Characterization of Phytochemicals and Polysaccharides in Diverse Coffee Cascara Samples: Identification, Quantification and Discovery of Novel Compounds

**DOI:** 10.3390/foods11121710

**Published:** 2022-06-10

**Authors:** Jiarong Zhang, Xuequan Sun, Pinhe Liu, Tongze Zhang, Joel A. Jelderks, Harold Corke

**Affiliations:** 1 Biotechnology and Food Engineering Program, Guangdong Technion-Israel Institute of Technology, Shantou 515063, China; jiarong.zhang@gtiit.edu.cn (J.Z.); lucasunluca@gmail.com (X.S.); pinhe.liu@gmail.com (P.L.); tongze.zhang@gtiit.edu.cn (T.Z.); 2 Caskai GmbH, Fuchsgasse 2, 4614 Marchtrenk, Austria; joel@caskai.com; 3 Faculty of Biotechnology and Food Engineering, Technion-Israel Institute of Technology, Haifa 3200003, Israel

**Keywords:** coffee cascara, valorization, phytochemicals characterization, novel compounds, pectic polysaccharides

## Abstract

Coffee cascara is the first and most significant by-product of the coffee processing industry, whose valorization has become an urgent priority to reduce harmful environmental impacts. This work aimed to provide an improved understanding of phytochemicals and polysaccharides in coffee cascara in order to offer information for the better evaluation of potential applications. Phytochemicals in 20 different coffee cascara samples were ultrasonically extracted and analyzed by HPLC-UV and HPLC-MS/MS. Four novel compounds were isolated for the first time from coffee cascara, including two still unknown tautomers (337 Da), and two dihydroflavonol glycosides (dihydromyricetin glycoside and dihydromyricetin rhamnosylglycoside). Their presence can contribute to the design of new value-added applications of coffee cascara. Chemical characterization of two polysaccharides from two of the coffee cascara pulp samples showed that they were mainly composed of homogalacturonan, with rhamnose and arabinose as minor neutral sugars. In addition, principal component analysis results indicated that coffee cultivar and/or country significantly impacted the phytochemical composition of coffee cascara, although differences may be reduced by the external environment and processing method. It is suggested that processing method should be carefully designed when generating coffee cascara from the same cultivar and country/farm.

## 1. Introduction

Coffee is a highly traded global commodity [1] and stands out as one of the most popularly consumed beverages worldwide, representing an important pillar of the economy in many developing countries [2]. In the coffee crop year of 2018/2019, the global output of coffee reached approximately 10 million tons [3]. However, it should be noted that this consumption only represents coffee beans, which account for just 20% of the total coffee berry weight. The remaining 80% corresponds to the by-products generated during coffee processing, which may be generally divided into two categories: pre-roasting by-products including coffee skin, pulp, mucilage, and parchment, and post-roasting by-product coffee silverskin [1,4]. Upon coffee harvesting and processing, cascara is the first generated by-product, whose characteristics and composition strongly depend on the post-harvest processing methods. Coffee cascara husk (CH) is obtained from the traditional dry method and comprises coffee skin, pulp, mucilage, parchment, and maybe part of the silverskin, accounting for nearly 45% of the total coffee cherry mass [5]. Coffee cascara pulp (CP) is commonly generated from the mechanical de-pulping process included in the wet method and semi-dry method [6], and mainly contains the coffee skin and pulp [7,8]. Therefore, coffee cascara husk and pulp are regarded as the two main by-products from coffee bean production, and a huge amount of these two by-products is generated every year. Generally, coffee cascara husk and pulp are considered environmental pollutants, and their accumulation will lead to severe environmental issues if they are not treated properly [9], especially with regard to the impact on freshwater ecosystems [10].

With the continuous increase of global coffee production and consumption, the coffee by-product management problem has become critical, and the development of novel applications based on the transformation of these by-products into value-added resources has received considerable interest from researchers worldwide in recent years, such as in biotechnological and agricultural/environmental applications, including the production of compost [11,12,13], mushrooms [14], enzyme [15], succinic acid [16], and biofuels [17,18,19]. In addition, coffee cascara (husk and pulp) has been verified as a safe food ingredient [20] and has noteworthy amounts of high-value components (such as protein, fibers, polysaccharides, and bioactive and flavor compounds) that could be recovered and reused as value-added products [21,22]. Therefore, food industrial applications of coffee cascara have also been developed [23]. 

Indeed, coffee cascara has been proven to be a potential source of macro- and/or micro-nutrients and non-nutrient health-beneficial bioactive compounds, which can be applied as either whole ingredients or specific-compound(s)-enriched extracts [7]. For instance, the extraction of sugars or flour from coffee cascara has been patented for use in products for human or animal consumption [24]. Moreover, the aqueous extract of coffee cascara has been applied in the production of healthy and sustainable yogurts possessing α-glucosidase inhibition activities [25], and a safe and novel “instant cascara” beverage with antioxidant properties has been developed based on this aqueous cascara extract [26]. Results from a comprehensive scientometric analysis showed that the most active applications of coffee by-products were based on the bioactive compounds, among which food applications were the most studied [27]. Therefore, it is possible to say that the coffee cascara-based formulation of functional food products is developing, is a research area of ongoing interest, and represents a feasible, efficient, and sustainable strategy for valorization of coffee cascara [27]. 

To design suitable processing strategies for such applications of coffee cascara, a proximate, quantitative characterization of all the phytochemicals is necessary and required but has been scarcely reported. In addition, the impact of the processing methods, cultivars, and cultivation areas of the coffee cascara samples should also be assessed, since they may affect in a significant manner the composition and concentration of constituents, and thus the proper utilization of the product [28]. 

In the present work, 20 coffee cascara (pulp and husk) samples generated from 13 cultivars were collected from 13 local farms in seven countries, and a preliminary characterization was carried out by HPLC-UV and HPLC-MS/MS, mainly including the identification and quantification of the main bioactive compounds and searching for novel compounds. Additionally, pectic polysaccharides present in two of the coffee pulp samples were also extracted and chemically characterized. The results obtained in this study will provide an improved understanding of phytochemicals and pectic polysaccharides in coffee cascara and may further contribute to the practical development/application of these coffee by-products as functional foods or beverages.

## 2. Materials and Methods

### 2.1. Materials and Chemicals

A total of 20 coffee cascara samples were collected and provided by Caskai GmbH, Marchtrenk, Austria, including 8 CP and 12 CH. These samples were all dried, and had average moisture contents of around 10%, and their detailed information is presented in Table 1. 

Commercial standards, including caffeine (≥98%), trigonelline hydrochloride (≥98%), protocatechuic acid (PA, ≥98%), gallic acid (≥98%), 3-caffeoylquinic acid (≥98%), xylose (Xyl, 99.04%), galactose (Gal, 99.07%), arabinose (Ara, 98.1%), Rhamnose (Rha, 98%), fructose (Fuc, 99.98%) and mannose (Man, 99.38%) were all purchased from Chendu RefMedic Biotech Co., Ltd. (Sichuan, China). Glucose (Glu, 99.8%), glucuronic acid (GluA, 99.1%), and galacturonic acid (GalA, 95.1%) standards were purchased from the National Institutes for Food and Drug Control (Beijing, China). NaOH (GR, 97%), Na_2_CO_3_ (AR, ≥99.5%), 3-phenylphenol (97%), phenol (AR), sodium tetraborate (99.5%), Folin-Ciocalteu’s phenol reagent (BR), and bovine serum albumin (BSA; BR) were obtained from Macklin Biochemical Co., Ltd. (Shanghai, China). Chloroform (CHCl_3_; AR, ≥99%), H_2_SO_4_ (AR, 95–98%), and acetic acid (AR, ≥99.5%) were obtained from Xilong Scientific (Shantou, China). Ethanol absolute (AR) was obtained from Guanghua Sci-Tech CO., Ltd. (Shantou, China). Bradford reagent was obtained from Bio-Rad (Hercules, CA, USA). ACS grade of formic acid (≥96%) and nitric acid (70%) were provided by Adamas and Aladdin (Shanghai, China), respectively. HPLC grade of methanol and trifluoroacetic acid (TFA, ≥99%) were obtained from Thermo Fisher Scientific (Waltham, MA, USA) and Sigma-Aldrich (St. Louis, MO, USA), respectively. 

### 2.2. Extraction and Identification of Phytochemicals in CP and CH Samples

CP and CH samples were ground into fine powder using a pulverizer (Xiamen Hehui Electronic Technology Co., Led., Fujian, China) and stored at 4 °C for further use. 

An ultrasonic-assisted method was used for extracting the phytochemicals in CP and CH. Briefly, the sample powder (1 g) was mixed with H_2_O (30 mL) at a solid-to-liquid ratio of 1:30 (*w*/*v*) and sonicated (Qsonica Q700-220 equipped with a probe tip of 12.7 mm diameter) for 3 min (1 s burst and 1 s cooling period) at 50% of maximal power. After sonication, the crude extract was centrifuged at 10,000× *g* (4 °C) for 20 min, and then the recovered supernatant was filtered through a 0.45 µm membrane, giving rise to the final sample, which was stored at 4 °C for further analysis. All the experiments were performed in triplicate.

HPLC analysis was performed on an Agilent 1220 Infinity II system, equipped with a diode array detector (DAD). Separation of the phytochemicals was carried out using a Shim-pack GIS C18 column (4.6 × 250 mm, 5 μm; Shimadzu), thermostated at 30 °C. The gradient elution program was as follows: 20% B at 0 min, 70% B at 70 min. The flow rate was 1 mL/min, and the injection volume was 10 µL. The detection wavelength was set at 280 and 320 nm, and on-line UV-vis spectra of the phytochemicals were recorded between 190 and 600 nm by means of the DAD. Solvent A was water with 0.1% formic acid (*v*/*v*) and solvent B was methanol with 0.1% formic acid (*v*/*v*). All the experiments were performed in triplicate.

HPLC elution fractions of the major phytochemicals were collected and then analyzed by electrospray ionization tandem mass spectrometry (ESI-MS/MS) for identification. Mass spectrometry (MS) was carried out using a TSQ Endura mass spectrometer (Thermo Fisher Scientific, Waltham, MA, USA) equipped with a heated electrospray ionization (HESI) source and a triple quadrupole analyzer. The fractions were infused into the ESI source at a flow rate of 0.16 mL/min by means of a UHPLC (UltiMate 3000, Thermo Fisher Scientific, Waltham, MA, USA) coupled with the mass spectrometer, and the solvent was methanol/H_2_O (50/50, *v*/*v*) acidified with 0.1% (*v*/*v*) formic acid. MS analyses were performed in both positive and negative modes under the following conditions: the ESI source temperature was 300 °C, the spray voltage was set at 3.5 kV for the positive mode and 3 kV for the negative mode, and nitrogen was used as sheath gas (35 Arbitrary, Arb) and aux gas (10 Arb). Mass spectra were recorded with a scan rate of 1000 Da/sec in the mass range *m*/*z* 100–1000. Tandem mass spectrometry analyses were performed with argon as collision-induced dissociation (CID) gas (0.5 m Torr) using a collision energy ramped from 0–55 V. The full-scan MS/MS spectra were obtained over an *m*/*z* range of 100–500. Data analysis was carried out using the Thermo Xcalibur software (version 4.0.27.10, Thermo Fisher Scientific, Waltham, MA, USA). 

### 2.3. Extraction of CP Pectic Polysaccharides (CPPs) 

CPPs were extracted according to the method previously described by Reichembach and de Oliveira Petkowicz [29], with minor modifications. Briefly, 3 g of the CP powder was boiled in 20 mL of 80% ethanol (*v*/*v*) for 20 min under reflux, in order to remove the pigments and low molecular weight compounds and inactivate the endogenous enzymes. The alcohol insoluble residue (AIR) was then recovered by vacuum filtration through a 0.45 µm membrane and washed with 10 mL of anhydrous ethanol three times. After drying at room temperature (18 °C), the AIR was mixed with 0.1 M HNO_3_ in a solid-liquid ratio of 1:25 (*w*/*v*) and boiled for 30 min under reflux to extract the CPPs. The mixture was filtered and concentrated at 10,000× *g* for 20 min, and the recovered supernatant was then used to precipitate the CPPs by addition of 2 volumes of anhydrous ethanol. After a 16-h precipitation at 4 °C, the CPPs were centrifuged (10,000× *g*, 20 min), washed with 10 mL of anhydrous ethanol three times, and lyophilized, giving the final CPP samples. 

### 2.4. Chemical Characterization of CPPs

#### 2.4.1. Determination of Protein, Total Phenolics, Total Polysaccharides and Uronic Acid Contents 

The content of protein, total phenolics, total polysaccharides, and uronic acid were all determined by colorimetric methods. The content of protein was measured by the Bradford method, and BSA was used as standard. The total phenolics content (TPC) was measured by the Folin–Ciolcateu method [30], and gallic acid was used as standard. The content of total polysaccharides was measured by the phenol-sulfuric acid method with glucose as standard. The content of uronic acid was measured by the *m*-hydroxydiphenyl method after saponification of the CPPs in 0.05 M NaOH for 30 min at room temperature [31], and GalA was used as standard. All the experiments were performed in triplicate. 

#### 2.4.2. Monosaccharide Composition

CPPs were hydrolyzed with 2 M TFA at 120 °C for 2 h, and the hydrolysates were then dried by rotary evaporation, washed three times with pure method to completely remove the TFA residue, and redissolved in H_2_O. After filtration through a 0.45 µm membrane, the samples were analyzed by HPLC (Agilent 1220 Infinity II equipped with a refractive index detector) using an Aminex^®^HPX-87H ion exclusion column (7.8 × 300 mm; Bio-Rad, Hercules, CA, USA). Monosaccharides were eluted isocratically with 5 mM H_2_SO_4_ under a flow rate of 0.45 mL/min. The injection volume was 20 µL, and the RID detector and the column were thermostated at 40 °C and 55 °C, respectively.

### 2.5. Statistical Analysis

Principal Component Analysis (PCA) was performed using SPSS Statistics 19.0 (IBM, Armonk, NY, USA) and visualized using Microsoft Office Excel Professional Plus 2019 software. The 7 quantified phytochemicals of 8 CP and 12 CH were analyzed by PCA, except for theobromine which was only found in CH sample 7.

## 3. Results

### 3.1. Identification and Quantification of Phytochemicals in CP Samples

**Figure 1:**
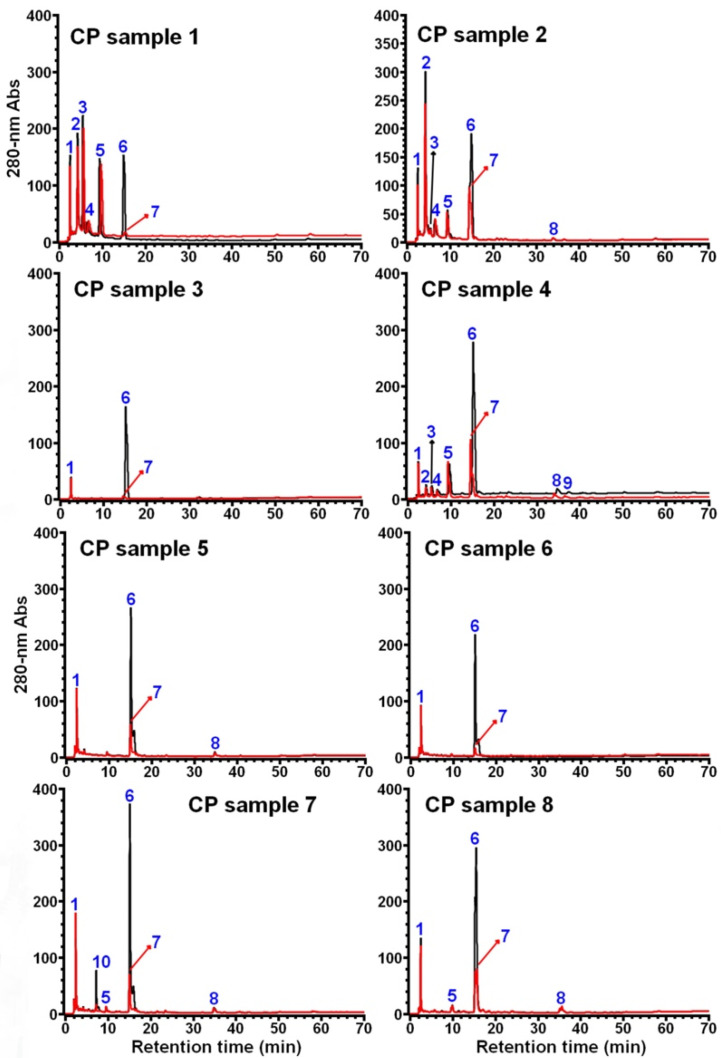
HPLC analysis of aqueous extract of 8 different CP samples. The chromatograms in black and in red were obtained based on a direct injection of the samples without (or before) and with (or after) CHCl_3_ treatment, respectively.

It was previously reported that coffee by-products contain a significant amount of caffeine [32], so HPLC analysis was carried out using both the aqueous CP extracts without (Figure 1, in black) and with (Figure 1, in red) chloroform (CHCl_3_) treatment (1:5, *v*/*v*), which could facilitate the identification of caffeine in the samples. 

HPLC analysis results showed that a total of 10 compounds (peaks 1–10, numbered following the sample order) were observed in all the 8 CP samples, and their identification was then performed by HPLC, MS (and/or MS/MS), and UV-vis spectrum analysis. 

**Figure 2:**
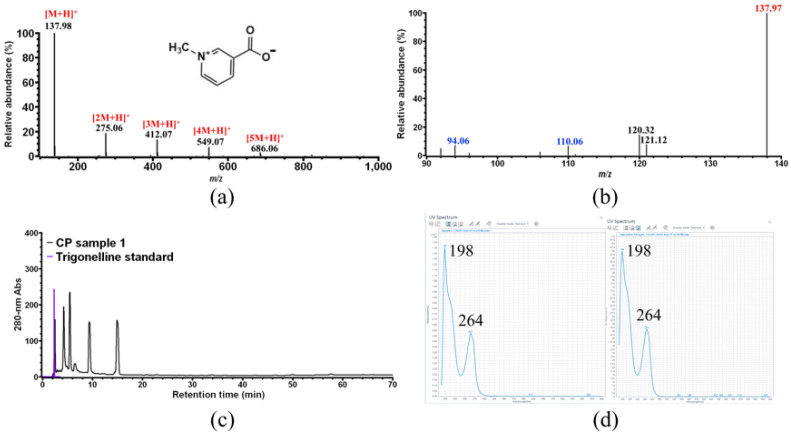
Identification of CP compound **1**. (**a**) MS analysis of compound **1**; (**b**) MS/MS analysis of the ion with *m*/*z* 137.98 obtained in (**a**); (**c**) HPLC overlay chromatogram of CP sample 1 and commercial trigonelline standard; (**d**) UV-vis spectrum analysis of compound **1** and trigonelline standard.

As shown in Figure 2a, five ions with *m*/*z* 137.98, 275.06, 412.07, 549.07, and 686.06 were observed on the MS spectrum of compound **1**. The predominant ion with *m*/*z* 137.98 corresponds to the [M + H]^+^ ion of compound **1**, whereas the other four minor ions are the [M + H]^+^ ion of dimer, trimer, tetramer, and pentamer of compound **1**. Further MS/MS analysis (Figure 2b) shows that the ion with *m*/*z* 137.98 is mainly fragmented into four ions with *m*/*z* 121.12, 120.32, 110.06, and 94.06. The MS and MS/MS information of compound **1** are quite similar with those of trigonelline [33]. Therefore, compound **1** was assigned to trigonelline, and its identification was further confirmed by HPLC (Figure 2c) and UV-vis spectrum (Figure 2d) using authentic trigonelline. 

**Figure 3:**
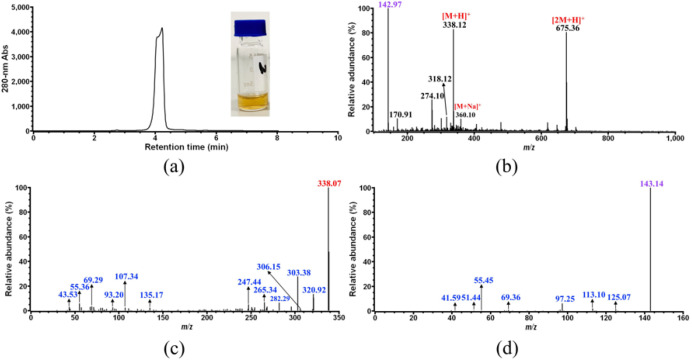
Identification of CP compound **2**. (**a**) HPLC analysis and photo of the concentrated sample of compound **2**; (**b**) MS analysis of the concentrated sample of compound **2**; (**c**) MS/MS analysis of the ion with *m*/*z* 383.12 obtained in (**b**); (**d**) MS/MS analysis of the ion with *m*/*z* 142.97 obtained in (**b**).

HPLC elution fraction of compound **2** was collected and directly analyzed by MS for identification. However, the [M + H]^+^ ion corresponding to compound **2** could not be distinguished from the background ions whose signal was strong due to low sample concentration. Therefore, an elution fraction of 100 mL was prepared, lyophilized, and redissolved in 1 mL pure methanol, giving a highly concentrated sample of compound **2**. As shown in Figure 3a, this concentrated sample only contains compound **2** at a much higher level than the original ones (Figure 1, CP sample 1, 2 and 4), and exhibits a yellow color (Figure 2b). Upon the second MS analysis of compound **2** (Figure 3b), three major ions with *m*/*z* 142.97, 338.12 and 675.36 were observed on the spectrum. It was most likely that the ion with *m*/*z* 338.12 corresponds to the [M + H]^+^ ion of compound **2**, because another major ion with *m*/*z* 675.36 should correspond to the [2M + H]^+^ ion derived from *m*/*z* 338, and the minor ion with *m*/*z* 360.10 observed in Figure 3b should correspond to the [M + Na]^+^ ion derived from *m*/*z* 338. Further MS/MS fragmentation of the [M + H]^+^ ion with *m*/*z* 338 gave two major ions with *m*/*z* 320.92 and 303.38 (Figure 3c). In addition, as shown in Figure 3c, several minor fragment ions with *m*/*z* less than 100 were also observed on the MS/MS spectrum of the ion with *m*/*z* 338, which were found on the MS/MS spectrum of the ion with *m*/*z* 142.97 as well (Figure 3d), indicating that the ion with *m*/*z* 142.97 obtained on Figure 3b was actually derived from *m*/*z* 338. Therefore, it could be concluded that the yellow-colored compound **2** would have a molecular weight of 337 Da, which could be fragmented into at least three ions with *m*/*z* 321, 303, and 143.

However, compound **2** still could not be successfully identified based on the MS and MS/MS data alone. 

**Figure 4:**
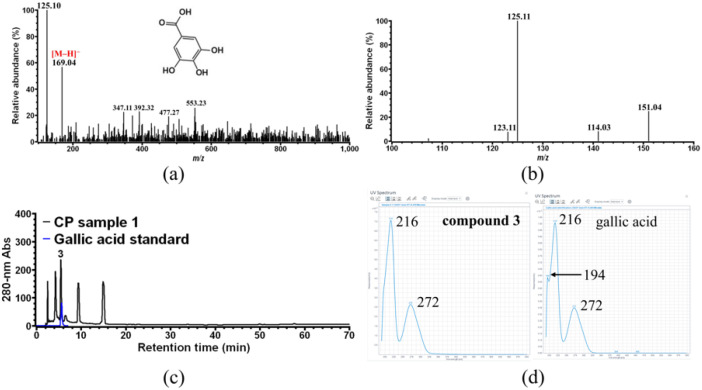
Identification of CP compound **3**. (**a**) MS analysis of compound **3**; (**b**) MS/MS analysis of the ion with *m*/*z* 169.04 obtained in (**a**); (**c**) HPLC overlay chromatogram of CP sample 1 and commercial gallic acid standard; (**d**) UV-vis spectrum analysis of compound **3** and gallic acid standard.

As shown in Figure 4a, two major ions with *m*/*z* 125.10 and 169.04 were observed on the MS spectrum of compound **3**. Further MS/MS of the latter (Figure 4b) shows that it is mainly broken down into one fragment ion with *m*/*z* 125.11, suggesting that the ion with *m*/*z* 125.10 observed in Figure 4a was already due to the fragmentation of *m*/*z* 169. Therefore, the ion with *m*/*z* 169 is the [M-H]^−^ ion of compound **3**, indicating that compound **3** would have a molecular weight of 170 Da, whose specific MS/MS fragment ion is that with *m*/*z* 125. These results were consistent with the reported MS and MS/MS data of gallic acid [34]. Finally, compound **3** was unambiguously identified as gallic acid by HPLC (Figure 4c) and UV-vis spectrum (Figure 4d) analysis using commercial gallic acid as the external standard. 

**Figure 5:**
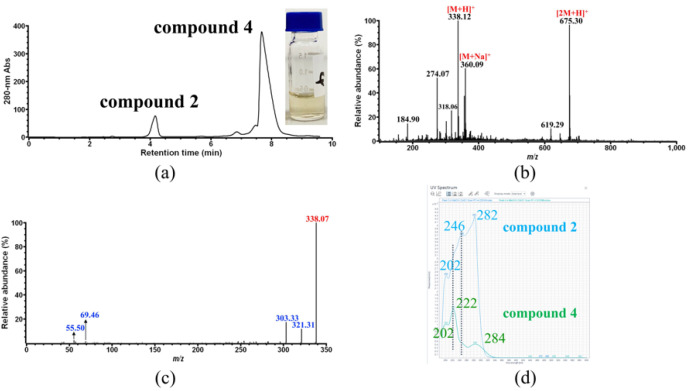
Identification of CP compound **4**. (**a**) HPLC analysis and photo of the concentrated sample of compound **4**; (**b**) MS analysis of the concentrated sample of compound **4**; (**c**) MS/MS analysis of the ion with *m*/*z* 383.12 obtained in (**b**); (**d**) Overlay UV-vis spectrum of the concentrated sample of compounds **2** and **4**.

According to the HPLC analysis (Figure 1), compound **4** was present in the samples at a very low concentration, so an elution fraction of 50 mL was collected by HPLC, which was then lyophilized and redissolved in 1 mL pure methanol, giving a more concentrated sample of compound **4** (Figure 5a) for use in further identification by MS. In addition, this concentrated sample also exhibits a light-yellow color, but it could not be concluded that compound **4** is itself yellow, because a certain level of contamination by compound **2** was also found in the sample (Figure 5a), which could contribute to the yellow color. 

As shown in Figure 5b, it was surprising that the obtained MS and MS/MS spectra of compound **4** are quite similar to those of compound **2** (Figure 3b,c), on the basis of which we could conclude that compound **4** would also have a molecular weight of 337 Da, the same as that of compound **2**.

It should be noted that compound **3** (gallic acid, peak 3) is eluted between compound **2** (peak 2) and compound **4** (peak 4) based on HPLC analysis (Figure 1, CP sample 1, in black). If the peak of contaminant compound **2** obtained in Figure 5a was introduced due to a poor manual collection of peak 4 during HPLC purification, then a peak corresponding to compound **3** should also be observed in Figure 5a, which is not the case. In addition, UV-vis spectrum analysis of the concentrated sample of compounds 2 and 4 (Figure 5d) showed that these two compounds have almost the same absorption behaviors in the 190–600 nm spectral range. Combined with the MS analysis results described above, this information indicated a strong relationship between compounds **4** and **2**, which could be concluded as follows: compound **4** is most likely a tautomer of compound **2**, which is less stable than the latter in solution and exists in an equilibrium of two tautomeric forms. Similarly, compound **4** also could not be identified based on the current data.

**Figure 6:**
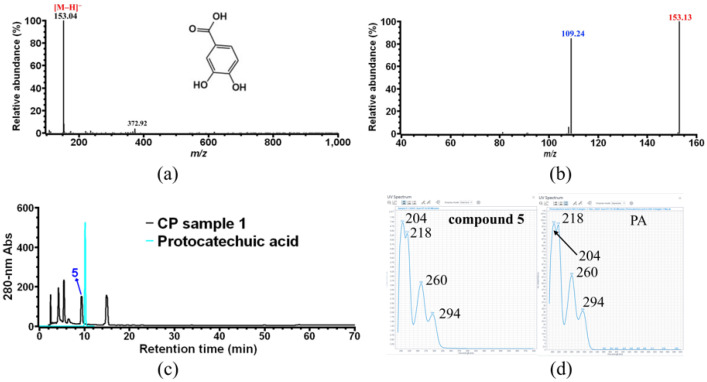
Identification of CP compound **5**. (**a**) MS analysis of compound **5**; (**b**) MS/MS analysis of the ion with *m*/*z* 153.04 obtained in (**a**); (**c**) HPLC overlay chromatogram of CP sample 1 and commercial PA standard; (**d**) UV-vis spectrum analysis of compound **5** and PA standard.

On the MS spectrum of compound **5** (Figure 6a), only one major ion with *m*/*z* 153.04 was observed, which corresponds to the [M–H]^−^ ion of compound **5**, and its MS/MS fragmentation only gives one ion with *m*/*z* 109.24 as a specific fragment ion (Figure 6b). It was previously reported that protocatechuic acid (PA) was found to be one of the main phenolics in coffee pulp, whose molecular weight is 154 Da [35]. Therefore, compound **5** was assigned to PA, and further confirmation was performed by HPLC (Figure 6c) and UV-vis spectrum analysis (Figure 6d) using authentic PA. 

**Figure 7:**
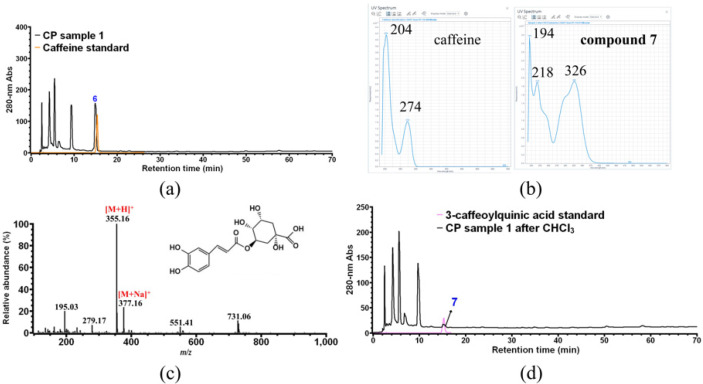
Identification of CP compounds **6** and **7**. (**a**) HPLC overlay chromatogram of CP sample 1 and commercial caffeine; (**b**) UV-vis spectrum analysis of caffeine standard and compound **7**; (**c**) MS analysis of compound **7**; (**d**) HPLC overlay chromatogram of 3-caffeoylquinic acid standard and CP sample 1 after CHCl_3_ treatment.

As shown in Figure 1, a significant decrease in the peak of compound **6** (in black) was observed after CHCl_3_ treatment (in red), indicating that compound **6** is most likely caffeine. As expected, compound **6** was assigned to caffeine after HPLC verification using the commercial caffeine standard (Figure 7a). Moreover, a small peak 7 at the HPLC retention time of caffeine was found after CHCl_3_ treatment, which would be caffeine residue or a new compound **7** that co-eluted with caffeine during HPLC analysis. As shown in Figure 7b, the UV-vis spectrum of peak 7 was distinct from that of caffeine, indicating that caffeine was completely removed by CHCl_3_ treatment, and peak 7 corresponds to a new compound **7** which cannot be detected in the presence of higher levels of caffeine. Finally, compound **7** was unambiguously identified as 3-caffeoylquinic acid (theoretical molecular weight of 354 Da) by HPLC-MS (Figure 7c) and HPLC (Figure 7d) analysis using commercial 3-caffeoylquinic acid as external standard.

**Figure 8:**
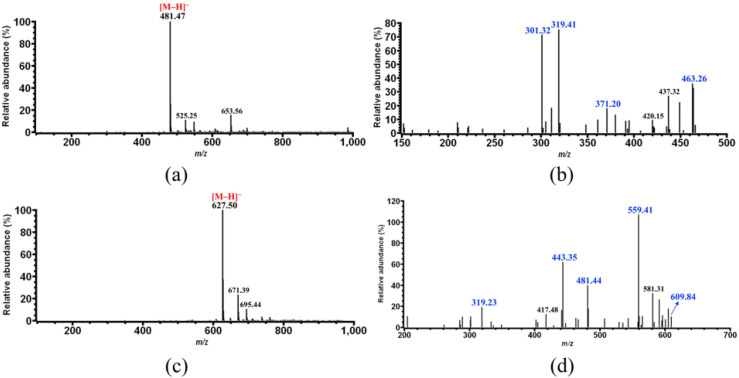
Identification of CP compounds **8** and **9**. (**a**) MS analysis of compound **8**; (**b**) MS/MS analysis of the ion with *m*/*z* 481.47 obtained in (**a**); (**c**) MS analysis of compound **9**; (**d**) MS/MS analysis of the ion with *m*/*z* 627.50 obtained in (**c**).

For compound **8**, only one major ion with *m*/*z* 481.47 was observed on its MS spectrum (Figure 8a), which certainly corresponds to the [M–H]^−^ ion of compound **8**. Further MS/MS fragmentation showed that this ion was broken down into several fragment ions with *m*/*z* 463.26, 437.32, 372.20, 319.41, and 301.32 (Figure 8b). Among these fragment ions, the one with *m*/*z* 463.26 was derived from the precursor ion with *m*/*z* 481.47 by water elimination (481 − 18 = 463 Da), and the one with *m*/*z* 319.41 could be obtained either from the precursor ion or the fragment ion with *m*/*z* 463 by loss of the glycosyl moiety (481 − 162 = 319 Da). This result suggested that compound **8** is most likely a glycosidic compound, and its specific MS/MS fragment ion is that with *m*/*z* 319, which also corresponds to the [M–H]^−^ ion of its aglycone (320 Da). Therefore, compound **8** was identified as dihydromyricetin glycoside. 

Compound **9** was only found in the CP sample 4 (Figure 1), whose molecular weight is 628 Da according to the MS analysis (Figure 8c). Although several fragment ions were observed on the MS/MS spectrum of compound **9** (Figure 8d), the presence of the ones with *m*/*z* 481 and 319 were more important for identification, which would indicate that compound **9** is most likely a dihydromyricetin glycoside (compound **8**) derivative. In addition, the fragment ions with *m*/*z* 609.84 and 481.44 would result from the precursor ion with *m*/*z* 627.50 by the loss of one water molecule (627 − 18 = 609 Da) and a rhamnosyl (627 − 146 = 481 Da), respectively, and further elimination of a second glycosyl from the latter ion would yield the ion with *m*/*z* 319.23 (481 − 162 = 319 Da). Therefore, compound **9** was identified as dihydromyricetin rhamnosylglycoside. 

**Figure 9:**
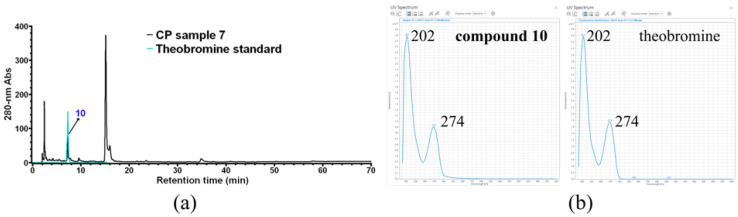
Identification of CP compound **10**. (**a**) HPLC overlay chromatogram of CP sample 7 and theobromine standard; (**b**) UV-vis spectrum analysis of compound **10** and theobromine standard.

Compound **10** was only observed in CP sample 7, which was unambiguously identified as theobromine based on HPLC and UV-vis spectrum analysis by using commercial theobromine as the external standard (Figure 9). 

In summary, compounds **1**, **3**, **5**, **6**, **7**, and **10** were unambiguously identified as trigonelline, gallic acid, protocatechuic acid, caffeine, 3-caffeoylquinic acid, and theobromine, respectively, including 3 alkaloids and 3 phenolic acids.

Compounds **2** and **4** were still unknown, but current data indicated that they were most likely two tautomers with molecular weight of 337 Da. As for compounds **8** and **9**, they were successfully identified as two dihydroflavonol glycosides, dihydromyricetin glycoside (DMG), and dihydromyricetin rhamnosylglycoside (DMRG); however, their exact structure determination requires further NMR analysis. 

In addition, the quantification and distribution of these 10 compounds in different CP samples is summarized in Table 2, and their identification information is also summarized in Table 3. 

### 3.2. Identification and Quantification of Phytochemicals in CH Samples

**Figure 10:**
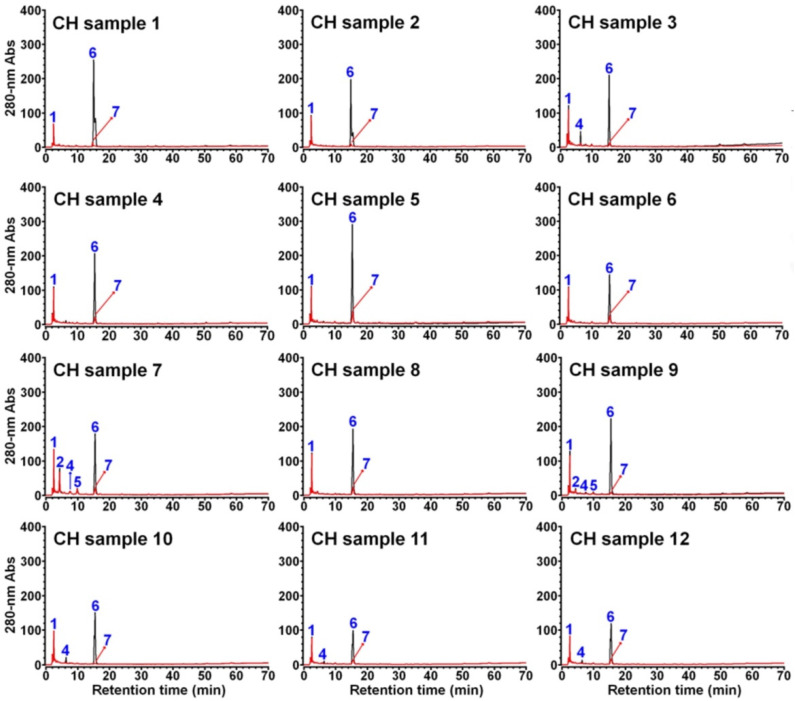
HPLC analysis of aqueous extract of 12 different CH samples. The chromatograms in black and in red were obtained based on a direct injection of the samples without (or before) and with (or after) CHCl_3_ treatment, respectively.

As shown in Figure 10, a total of 6 compounds were observed in the 12 CH samples, all of which were already found in the CP samples, including already unambiguously identified trigonelline (compound **1**), protocatechuic acid (compound **5**), caffeine (compound **6**), and 3-caffeoylquinic acid (compound **7**), as well as the two still unknown tautomers (compounds **2** and **4**), and their quantification and distribution in different CH samples is summarized in Table 4. 

In general, CH samples contained fewer compounds compared to CP samples. Because gallic acid, theobromine, and the two novel dihydromyricetin glycosides (compounds **8** and **9**) were not observed in CH samples, they may not exist at all or are present in an undetectable level. Therefore, it could be considered if these four compounds might be further used as biological marker compounds for identification of some coffee cultivars or coffee cascara samples. Moreover, for the 4 common compounds previously reported in coffee cascara samples, including trigonelline, caffeine, 3-caffeoylquinic acid, and protocatechuic acid, the average value of the three former compounds in CP samples (trigonelline: 6.84 mg/g; caffeine: 7.07 mg/g; 3-caffeoylquinic acid: 3.96 mg/g) were higher than in CH samples (trigonelline: 6.49 mg/g; caffeine: 5.34 mg/g; 3-caffeoylquinic acid: 1.12 mg/g), and the latter was scarcely present in CH samples. 

It is also worth noting that the identification and quantification of 3-caffeoylquinic acid in coffee cascara samples should be carefully performed, because it could not be separated from caffeine under some HPLC gradients and would then be easily ignored in the presence of high levels of caffeine. Thus, at least two detection wavelengths were proposed during HPLC analysis, including 280 nm and 360 nm, since caffeine does not have any absorption at 360 nm. 

### 3.3. Chemical Characterization of CPPs

As described above, more compounds were found in the two CP samples 1 (*Coffea arabica* L. var. *Castillo*) and 2 (Wush Wush, Ethiopian heirloom) from Colombia, whose contents were also relatively higher, showing a better potential for further application from the point view of bioactive compounds. Moreover, coffee pulp was reported as a source of pectic polysaccharides, and a more recent study on a pectin from Brazilian coffee (*Coffea arabica* L.) pulp suggested the role of coffee pectin as a suitable ingredient in the food industry [29]. Therefore, these two CP samples were finally selected for CPP extraction and preliminary characterization, which may be used for further evaluation of their application potential from the point view of macromolecular compounds. 

Generally, as shown in Table 5, CPPs from CP sample 1 (CPPs-1) and CP sample 2 (CPPs-2) contained similar levels of protein and total phenolics. Furthermore, CPPs-1 was obtained in a higher yield than CPPs-2, while its galacturonic acid content was lower than that of CPPs-2. 

**Figure 11:**
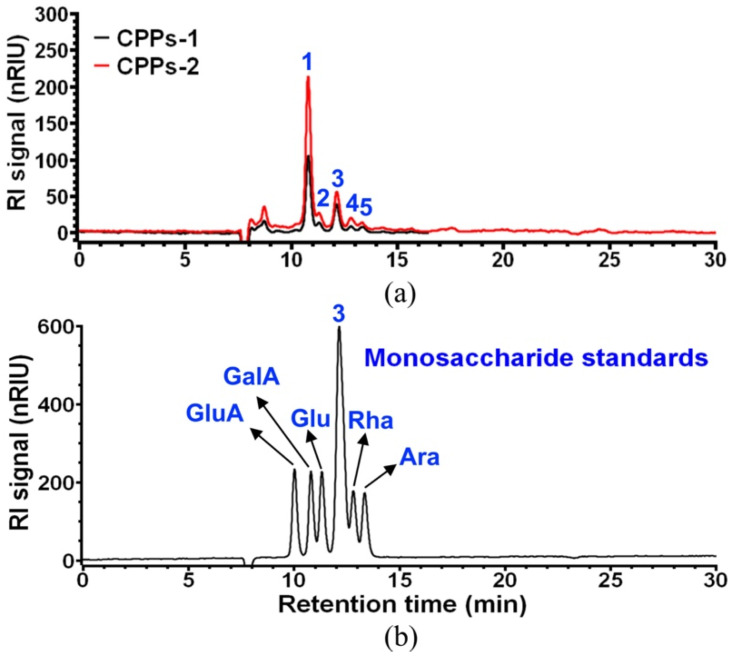
HPLC analysis of constituent monosaccharides of CPPs-1 and CPPs-2 (**a**) and monosaccharide standards (**b**). The peaks 1, 2, 4 and 5 in (**a**) are identified as GalA, Glu, Rha and Ara, respectively. Peak 3 in (**a**) cannot be identified, because its corresponding standard peak (blue peak 3 in (**b**)) represents the peak of co-eluted Gal, Xyl, Man and Fuc.

It was reported that the composition of pectic polysaccharides varies according to the plant source and extraction methods [36], and galacturonic acid is predominantly contained in pectic polysaccharides as residues of their main linear chains (homogalacturonan or HG segments, also known as ‘smooth regions’), as well as other neutral sugars in the side chains (rhamnogalacturonans or RG-I segments, also known as ‘hair regions’) [37], such as rhamnose, arabinose, galactose, etc. [38]. Therefore, monosaccharides determine the unique structures and properties of polysaccharides as natural basic units, and their composition is required for the structural characterization of polysaccharides [39]. The results showed that CPPs-1 and CPPs-2 had similar pectic monosaccharide compositions, suggesting that the effect of cultivar on the composition of polysaccharides might be reduced when the external environment was the same, since these two CP samples were collected from the same local farm in Colombia. Of course, an originally similar polysaccharide composition of these two coffee cultivars still could not be fully excluded. As shown in Figure 11 and Table 6, GalA was measured as the predominant constituent monosaccharide of both CPPs-1 and CPPs-2, and Glu, Rha, and Ara were their minor neutral constituent monosaccharides. However, the main neutral monosaccharide (visualized as peak 3 in Figure 11a) of these two CPP samples still could not be confirmed, because the Aminex^®^HPX-87H ion exclusion column could not separate Gal, Xyl, Man, and Fuc, and other methods such as HPAEC (High performance anion exchange chromatography), gas chromatography (GC), and GC-mass spectrometry (GC-MS) should be considered for a complete characterization. Thus, these results suggested that CPPs-1 and CPPs-2 could mainly consist of HG, meaning polygalacturonic acid-rich ‘smooth regions’, but their side chain lengths that can be revealed by the value of the molar ratio (Ara + Gal)/Rha [29] could not be estimated due to a lack of the molar proportion of Gal. 

## 4. Discussion

### 4.1. PCA Analysis of 20 Coffee Cascara Samples

**Figure 12:**
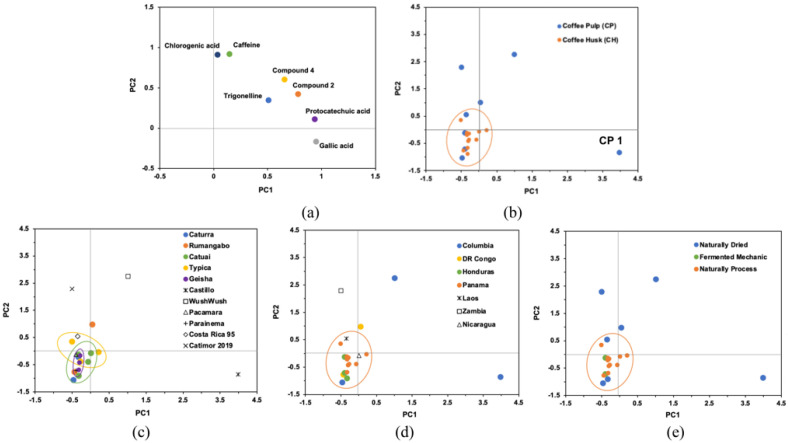
Principal component analysis (PCA) applied to the distribution of phytochemicals (**a**) and coffee cascara pulp and husk samples (**b**), and the visualization of the impact of coffee cultivar (**c**), country (**d**) and process (**e**).

Principal component analysis was conducted on seven phytochemicals among 20 coffee cascara samples (Figure 12). The first two principal components, PC1 (44.3%) and PC2 (34.0%), explained 78.3% of the total variance. These seven phytochemicals (Figure 12a) determined the distribution of the 20 coffee cascara samples (Figure 12b), among which caffeine and 3-caffeoylquinic acid had greater impact. Compared to CP samples, relative lower content of caffeine and 3-caffeoylquinic acid within a small variation range (Table 2 and Table 4) led to a good clustering of CH samples (Figure 12b). CP samples did not present a clustering, mainly because they were generated from distinct coffee cultivars (due to a random sample collection) (Figure 12c) and collected from different countries (Figure 12d), which is why their content of caffeine and 3-caffeoylquinic acid varied in a significant manner. Therefore, these analyses implied that coffee cultivar and/or country determined the phytochemical composition of coffee cascara. 

In addition, although CH samples were also produced from five different coffee cultivars, they still clustered (Figure 12c), which indicates their similarities in phytochemicals. This was most likely because 4 of the coffee cultivars were collected from the same country (Panama, Figure 12d), and the same process method (natural process) was applied (Figure 12e), which could suggest that the impact of coffee cultivar on the phytochemicals might be reduced by the same external environments, such as country (mainly geography, climate, and soil) and process method. This also implied that when coffee cascara was generated from the same cultivar and country, the selection of process method would be important. Of course, it still could not be fully excluded that these four coffee cultivars (Caturra, Typica, Geisha, and Catuai) were originally similar in phytochemicals. 

However, a more substantial evaluation was limited by the number of biological replicates investigated. 

### 4.2. Possible Structure of the Four Unknown Compounds

As described above, unknown compounds **2** and **4** were most likely two tautomers with molecular weight of 337 Da, which might be considered as derivatives of *p*-coumaroylquinic acid according to several excellent studies on in-depth characterization of chlorogenic acids by LC-MS^n^ [40,41,42,43]. To the best of our knowledge, they were isolated for the first time from coffee cascara, which could be therefore regarded at least as novel coffee cascara compounds. However, their possible structure could not be predicted in this study due to a lack of sufficient MS/MS fragmentation information. The reason was that their corresponding [M + H]^+^ ion with *m*/*z* 338 could not be well fragmented at low collision energy (Figure 3c and Figure 5c), and this ion was completely fragmented when the energy was increased slightly, leading to an observation of fragment ions mainly with *m*/*z* less than 100 (data not shown). Therefore, their specific MS/MS fragment ions could not be clarified in both cases, which were important for preliminary prediction/identification of their structure. Further optimization of the MS/MS analytical conditions should be conducted, or other analysis methods should be considered. It was found that the novel compound **2** was present in a significant amount in two CP samples (1 and 2, Castillo and Wush Wush cultivars from Colombia) and one CH sample (7, Typica cultivar from Panama), and further clarification of its structure and bioactivities would be beneficial for developing new value-added applications of these coffee cascara in the food, beverage, or cosmetics industries. 

Dihydroflavonols represent a relatively scarce group of flavonoids [44], which serve as substrates for the production of colored anthocyanins and flavonols in the biosynthetic pathway of flavonoids. In the reported dihydroflavonol glycosides, most of them were identified as glycosides derived from dihydrokaempferol and dihydroquercetin. However, dihydromyricetin glycosides and dihydromyricetin disaccharides were rarely reported, so only a few examples were found, such as dihydromyricetin-3-O-rhamnoside naturally isolated from the leaves of *Erica arborea* (Ericaceae) [45], and dihydromyricetin diglucoside and its methylated derivative from jambolão (*Syzygium cumini*) [46]. For coffee cascara, the previous studies were mostly focused on several well-known compounds, such as caffeoylquinic acids, caffeine, trigonelline, and protocatechuic acid [32,47]. Recently, a study on in-depth characterization of the bioactive compounds in coffee silverskin was reported, in which 30 compounds were identified [48]. However, such dihydroflavonol glycosides were still not found. Therefore, the two dihydromyricetin glycosides (compounds **8** and **9**) observed in this study could be regarded as novel coffee cascara polyphenolic compounds, which were reported in coffee cascara for the first time. 

**Figure 13:**
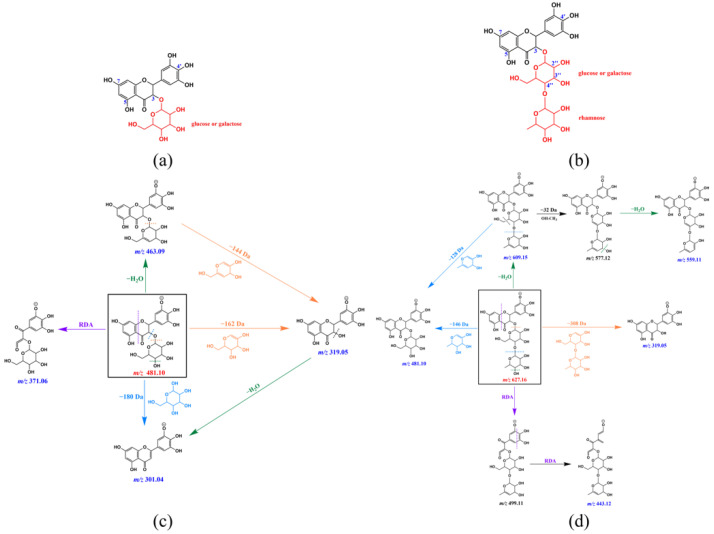
Possible structure and MS/MS fragmentation pathway of novel CP compounds **8** (**a**,**c**) and **9** (**b**,**d**). The site of deprotonation in [M–H]^−^ was randomly selected, and RDA is short for Retro-Diels-Alder fragmentation. The precursor or parent ions are present in the box, and the dotted lines with different color represent different ways of fragmentation. The position of the rhamnosyl group of the compound **9** in (**b**) and (**d**) was randomly selected among C_2__″_, C_3__″_ and C_4__″_.

Generally, glycosylation was observed at the C_3_-OH of the benzopyran ring C for flavonols and dihydroflavonols. Therefore, these two compounds could be tentatively assigned to dihydromyricetin 3-O-glycoside and dihydromyricetin-3-O-rhamnosyl-glycoside (glycosylation at C_3_-OH of the benzopyran ring C, Figure 13a,b), respectively, and their possible MS/MS fragmentation pathways were also established based on the MS and MS/MS data (Figure 13c,d). However, the glycosylation at C_5_- and C_7_-OH of the benzopyran ring A and at the C_4′_-OH of the aromatic ring B could not be fully excluded, since glycosylation of dihydrokaempferol and dihydroquercetin at these positions has been reported, such as dihydrokaempferol-5-O-glucoside (Helicioside A) and dihydroquercetin-5-O-glucoside (Helicioside B) from the leaves of *Helicia cochinchinensis* (Proteaceae) [49], dihydroquercetin-7-O-glucoside [50], and dihydroquercetin-4′-O-glucoside [51]. In addition, the current MS and MS/MS data could not support a clear identification of the glycosyl group (glucose or galactose) of these two compounds, as well as the interglycosidic linkage between the rhamnosyl and glycosyl group (at C_2″_, C_3″’_ or C_5″_ of the glycosyl group, Figure 13b) for the dihydromyricetin-3-O-rhamnosylglycoside, and further identification by NMR analysis is required. 

It is worth noting that a Chinese study has reported that an enzymatically synthesized dihydromyricetin-7-O-glucoside showed quite similar antidiabetic activity with the antidiabetic drug gliquidone during a clinical trial [52]. Therefore, further detailed structure and bioactivity investigation of these two novel compounds should be performed. 

## 5. Conclusions

Phytochemicals from 20 coffee cascara samples (eight coffee cascara pulps and 12 coffee cascara husks) were analyzed by HPLC-UV and HPLC-MS/MS. A total of 10 compounds were observed in eight coffee cascara pulp samples, among which six were also found in 12 coffee cascara husk samples. After identification, six of the 10 compounds were unambiguously identified, including three alkaloids (trigonelline, caffeine, and theobromine) and three phenolic acids (gallic acid, protocatechuic, acid and 3-caffeoylquinic acid). The other four compounds were isolated for the first time from coffee cascara, which could be therefore regarded as novel coffee cascara compounds, including two still unknown tautomers with molecular weight of 337 Da, and two flavonoid glycosides, dihydromyricetin glycoside and dihydromyricetin rhamnosylglycoside. The observation of these compounds would provide an improved understanding of the phytochemicals composition of coffee cascara and may contribute to the design of new value-added application of these coffee by-products in the nutraceutical, cosmetics, or even pharmaceutical industries. 

In addition, pectic polysaccharides were also extracted from two of the coffee cascara pulp samples and characterized. The results showed that they mainly consisted of polygalacturonic acid-rich ‘smooth regions’. However, their clear neutral sugar composition and degrees of methylation and acetylation should be further determined, which would provide more information for estimating the application potentials of coffee cascara pulp from the point view of macromolecules. 

## Figures and Tables

**Table 1:** Detailed information of coffee cascara samples.

	No.	Country	Farm	Cultivar	Process
CP	1	Colombia	EI Zafiro	Castillo	Naturally Dried
	2	Colombia	EI Zafiro	Wush Wush	Naturally Dried
	3	Colombia	EI Zafiro	Caturra	Naturally Dried
	4	DR Congo	Virunga Café	Rumangabo	Naturally Dried
	5	Honduras	EI Parasio	Pacamara	Fermented Mechanically Dried
	6	Honduras	EI Parasio	Parainema	Fermented Mechanically Dried
	7	Laos	Thevada Estate	Costa Rica 95	Naturally Dried
	8	Zambia	Kateshi Estate	Catimor 2019	Naturally Dried
CH	1	DR Congo	Virunga Café	Rumangabo	Natural Process
	2	Honduras	EI Paraiso	Catuai (Red)	Natural Process
	3	Nicaragua	St Maria Lourdes	Catuai	Natural Process
	4	Panama	Carmen Estate	Caturra	Natural Process
	5	Panama	Hacienda Bajo Mono	Typica	Natural Process
	6	Panama	Elida Estate	Geisha	Natural Process
	7	Panama	Elida Estate	Typica	Natural Process
	8	Panama	Elida Estate	Catuai	Natural Process
	9	Panama	EI Burro Estate	Geisha	Natural Process
	10	Panama	Casa Ruiz	Catuai	Natural Process
	11	Panama	Casa Ruiz	Geisha	Natural Process
	12	Panama	Berlina Estate	Typica	Natural Process

**Table 2:** Quantification and distribution of the 10 CP compounds (mg/g).

CP No.	Trigonelline	Compound 2 ^†^	Gallic Acid	Compound 4 ^†^	Protocatechuic Acid	Caffeine	3-caffeoylquinic Acid ^‡^	Theobromine	DMG	DMRG
1	10.53 ± 2.30 ^a^	1.85 ± 0.018 ^b^	6.97 ± 0.026 ^a^	0.15 ± 0.023 ^b^	9.79 ± 0.131 ^a^	6.30 ± 0.060 ^e^	0.65 ± 0.036 ^f^	-	-	-
2	7.19 ± 0.019 ^b^	2.48 ± 0.013 ^a^	0.20 ± 0.015 ^c^	0.23 ± 0.01 ^a^	3.34 ± 0.161 ^c^	10.69 ± 0.426 ^b^	8.58 ± 0.289 ^a^	-	+	-
3	2.94 ± 0.012 ^d^	-	-	-	0.02 ± 0.0 ^d^	3.21 ± 0.281 ^h^	0.52 ± 0.002 ^f^	-	-	-
4	4.38 ± 0.116 ^c^	0.17 ± 0.009 ^c^	0.48 ± 0.014 ^b^	0.07 ± 0.003 ^d^	4.43 ± 0.077 ^b^	6.84 ± 0.523 ^d^	8.21 ± 0.125 ^b^	-	+	+
5	6.63 ± 0.069 ^b^	-	-	-	-	5.45 ± 0.135 ^f^	2.53 ± 0.04 ^e^	-	+	-
6	4.72 ± 0.02 ^c^	-	-	-	-	4.39 ± 0.045 ^g^	0.72 ± 0.063 ^f^	-	-	-
7	9.17 ± 0.025 ^a^	-	-	-	-	7.51 ± 0.067 ^c^	3.47 ± 0.324 ^d^	1.06 ± 0.002	+	-
8	9.16 ± 0.111 ^a^	-	-	0.09 ± 0.001 ^c^	-	12.18 ± 0.055 ^a^	6.96 ± 0.120 ^c^	-	+	-

Values are expressed as the mean ± SD. Different letters within same columns indicate significant difference at *p* < 0.05. “^†^”: quantified by using the standards prepared in lab, and the others were quantified based on the use of authentic standards; “-”: not detectable; “+”: compounds detected in the sample but unquantifiable due to lack of authentic standards; “^‡^”: quantified based on its absorption at 360 nm, where caffeine doesn’t have any absorption.

**Table 3:** Identification of the 10 compounds in CP samples by HPLC, MS and MS/MS.

Compound No.	RT * (min)	λmax (nm)	MW	[M + H]^+^/[M–H]^−^	MS/MS Fragment Ions	Identification
1	2.5	198, 264	137	137.98/-	121.12, 120.32, 110.06, 94.06	Trigonelline
2	4.3	202, 246, 282	337	338.12/-	320.92, 303.38, 142.97	Unknown
3	5.5	194, 216, 272	170	-/169.04	151.04, 114.03, 125.11	Gallic acid
4	6.6	202, 222, 284	337	338.12/-	321.31, 303.33	Unknown
5	9.7	204, 218, 260, 294	154	-/153.04	109.24	Protocatechuic acid
6	14.9	204, 274	194	-/-	-	Caffeine
7	14.9	194, 218, 326	354	355.16/-	-	3-caffeoylquinic acid
8	35	-	482	-/481.47	463.26, 371.20, 319.41, 301.32	Dihydromyricetin glycoside
9	38	-	628	-/627.50	559.41, 481.44, 443.35, 319.23	Dihydromyricetin rhamnosylglycoside
10	7.3	202, 274	180	-/-	-	Theobromine

“*”: HPLC retention time, RT; “-”: not measured.

**Table 4:** Quantification and distribution of the 6 CH compounds (mg/g).

CH No.	Trigonelline	Compound 2 ^†^	Compound 4 ^†^	Protocatechuic Acid	Caffeine	3-Caffeoylquinic Acid ^‡^
1	4.06 ± 0.071 ^g^	-	-	-	4.44 ± 0.638 ^f^	0.53 ± 0.038 ^ef^
2	5.04 ± 0.039 ^f^	-	-	-	3.63 ± 0.127 ^g^	0.35 ± 0.015 ^f^
3	7.28 ± 0.038 ^bc^	-	0.11 ± 0.003 ^a^	-	5.36 ± 0.079 ^e^	0.75 ± 0.08 ^e^
4	7.02 ± 0.074 ^c^	-	-	-	5.87 ± 0.108 ^cd^	1.41 ± 0.071 ^cd^
5	6.54 ± 0.054 ^d^	-	-	-	8.08 ± 0.328 ^a^	2.43 ± 0.342 ^a^
6	6.85 ± 0.137 ^cd^	-	-	-	4.52 ± 0.122 ^f^	1.71 ± 0.228 ^b^
7	7.89 ± 0.41 ^a^	0.53 ± 0.038	0.05 ± 0.005 ^b^	1.24 ± 0.09	5.65 ± 0.363 ^de^	1.47 ± 0.109 ^bcd^
8	7.26 ± 0.211 ^bc^	-	-	-	6.10 ± 0.109 ^c^	1.64 ± 0.179b ^c^
9	7.54 ± 0.033 ^ab^	-	+	+	6.77 ± 0.097 ^b^	0.61 ± 0.047 ^e^
10	7.73 ± 0.147 ^a^	-	0.05 ± 0.008 ^b^	-	5.26 ± 0.127 ^e^	+
11	4.82 ± 0.126 ^f^	-	0.03 ± 0.001 ^d^	-	3.68 ± 0.093 ^g^	1.21 ± 0.097 ^d^
12	5.83 ± 0.61 ^e^	-	0.04 ± 0.0 ^c^	-	4.68 ± 0.128 ^f^	1.37 ± 0.051 ^d^

Values are expressed as the mean ± SD. Different letters within same columns indicate significant difference at *p* < 0.05. “^†^”: quantified by using the standards prepared in lab, and the others were quantified based on the use of authentic standards; “-”: not detectable; “+”: compound detected in the sample but unquantifiable due to very low concentration; “^‡^”: quantified based on its absorption at 360 nm, where caffeine doesn’t have any absorption.

**Table 5:** Yield and chemical composition of CPPs-1 and CPPs-2.

	CPPs-1	CPPs-2
Yield * (%)	10.3%	7.1%
Protein (%)	3.49 ± 0.23	3.26 ± 0.06
Total phenolics (%)	1.34 ± 0.23	0.89 ± 0.08
Total GalA (%)	69.66 ± 5.42	79.20 ± 4.02

Values are expressed as the mean ± SD. * CPPs yield was calculated according to dry mass of the AIR using the following equation: Yield (%) = (mass of lyophilized CPPs/mass of dried AIR) × 100.

**Table 6:** Monosaccharide composition and their molar ratio of CPPs-1 and CPPs-2.

	Monosaccharides and Molar Ratios					
	GluA	GalA	Glu	Rha	Ara	Gal/Xyl/Man/Fuc
CPPs-1	0.04	2.09	0.23	0.27	0.26	nc
CPPs-2	nd	4.19	0.36	0.31	0.59	nc

“nd”: not detectable; “nc”: not confirmed, due to a poor separation of Gal, Xyl, Man and Fuc as described above.

## Data Availability

Data reported and analyzed in this paper have been archived in laboratory notebooks and computer files at GTIIT, Guangdong Technion-Israel Institute of Technology.

## References

[1] Tucker C.M., Tucker C.M. (2017). Theories of food and social meanings of coffee. Coffee Culture: Local Experiences, Global Connections.

[2] Gerz A., Avelino J., van de Kop P., Sautier D., Gerz P. (2006). Costa rican arabica coffee: Legitimacy of for specialty. Origin-Based Products: Lessons for Pro-Poor Market Development.

[3] Hejna A. (2021). Potential applications of by-products from the coffee industry in polymer technology-current state and perspectives. Waste Manag..

[4] Lopes A.C.A., Andrade R.P., de Oliveira L.C.C., Lima L.M.Z., Santiago W.D., de Rescende M.L.V., das Graças Cardoso M., Duarte W.F. (2020). Production and characterization of a new distillate obtained from fermentation of wet processing coffee by-products. J. Food Sci. Technol..

[5] Del Castillo M.D., Fernandez-Gomez B., Martinez-Saez N., Iriondo-DeHond A., Mesa M.D., Farah A. (2019). Coffee by-products. Coffee: Production, Quality and Chemistry.

[6] Bonilla-Hermsa V.A., Duarte W.F., Schwan R.F. (2014). Utilization of coffee by-products obtained from semi-washed process for production of value-added compounds. Bioresour. Technol..

[7] Iriondo-DeHond A., Iriondo-DeHond M., Del Castillo M.D. (2020). Applications of compounds from coffee processing by-products. Biomolecules.

[8] Mussatto S.I., Machado E.M.S., Martins S., Teixeira J.A. (2011). Production, composition, and application of coffee and its industrial residues. Food Bioprocess Technol..

[9] Janissen B., Huynh T. (2018). Chemical composition and value-adding applications of coffee industry by-products: A review. Resour. Conserv. Recycl..

[10] Awoke A., Beyene A., Kloos H., Goethals P.L.M., Triest L. (2016). River water pollution status and water policy scenario in Ethiopia: Raising awareness for better implementation in developing countries. Environ. Manag..

[11] Dzung N.A., Dzung T.T., Khanh V.T.P. (2013). Evaluation of coffee husk compost for improving soil fertility and sustainable coffee production in rural central highland of Vietnam. Resour. Environ..

[12] Figueroa G.A., Homann T., Rawel H.M. (2016). Coffee production wastes: Potentials and perspectives. Austin Food Sci..

[13] Ulsido M.D., Li M. (2016). Effect of organic matter from coffee pulp compost on yield response of chickpeas (*Cicer arietinum* L.) in Ethiopia. Eng. Rural Develop..

[14] da Silva M.C.S., Naozuka J., da Luz J.M.R., de Assunção L.S., Oliveira P.V., Vanetti M.C.D., Bazzolli D.M.S., Kasuya M.C.M. (2012). Enrichment of *Pleurotus ostreatus* mushrooms with selenium in coffee husks. Food Chem..

[15] Navya P.N., Murthy Pushpa S. (2013). Production, statistical optimization and application of endoglucanase from *Rhizopus stolonifera* utilizing coffee husk. Bioprocess Biosyst. Eng..

[16] Dessie W., Zhu J., Xin F., Zhang W., Jiang Y., Wu H., Ma J., Jiang M. (2018). Bio-succinic acid production from coffee husk treated with thermochemical and fungal hydrolysis. Bioprocess Biosyst. Eng..

[17] Jayachandra T., Venugopal C., Anu Appaiah K.A. (2011). Utilization of phytotoxic agro waste-coffee cherry husk through pretreatment by the ascomycetes fungi *Mycotypha* for biomethanation. Energy Sustain. Dev..

[18] Menezes E.G.T., do Carmo J.R., Alves J.G.L.F., Menezes A.G.T., Guimarães I.C., Queiroz F., Pimenta C.J. (2014). Optimization of alkaline pretreatment of coffee pulp for production of bioethanol. Biotechnol. Prog..

[19] Woldesenbet A.G., Woldeyes B., Chandravanshi B.S. (2016). Bio-ethanol production from wet coffee processing waste in Ethiopia. SpringerPlus.

[20] Iriondo-DeHond A., Aparicio García N., Fernandez-Gomez B., Guisantes-Batan E., Velázquez Escobar F., Blanch G.P., San Andres M.I., Sanchez-Fortun S., del Castillo M.D. (2019). Validation of coffee by-products as novel food ingredients. Innov. Food Sci. Emerg. Technol..

[21] Dorsey B.M., Jones M.A., Galanakis C.M. (2017). Healthy components of coffee processing by-products. Handbook of Coffee Processing By-Products: Sustainable Applications.

[22] Baiano A. (2014). Recovery of biomolecules from food wastes—A review. Molecules.

[23] Klingel T., Kremer J.I., Gottstein V., de Rezende T.R., Schwarz S., Lachenmeier D.W. (2020). A review of coffee by-products including leaf, flower, cherry, husk, silver skin, and spent grounds as novel foods within the European Union. Foods.

[24] Velez A.R., Lopez J.C.J. (2017). Process for obtaining honey and/or flour of coffee from the pulp or husk and the mucilage of the coffee bean. U.S. Patent.

[25] Iriondo-DeHond M., Iriondo-DeHond A., Herrera T., Fernández-Fernández A.M., Sorzano C.O.S., Miguel E., del Castillo M.D. (2020). Sensory acceptance, appetite control and gastrointestinal tolerance of yogurts containing coffee-cascara extract and inulin. Nutrients.

[26] Iriondo-DeHond A., Elizondo A.S., Iriondo-DeHond M., Ríos M.B., Mufari R., Mendiola J.A., Ibañez E., del Castillo M.D. (2020). Assessment of healthy and harmful maillard reaction products in a novel coffee cascara beverage: Melanoidins and acrylamide. Foods.

[27] Durán-Aranguren D.D., Robledo S., Gomez-Restrepo E., Arboleda Valencia J.W., Tarazona N.A. (2021). Scientometric overview of coffee by-products and their applications. Molecules.

[28] Elías L.G., Braham J.E., Bressani R. (1979). Chemical composition of coffee-berry by-products. Coffee Pulp: Composition, Technology, and Utilization.

[29] Reichembach L.H., de Oliveira Petkowicz C.L. (2020). Extraction and characterization of a pectin from coffee (*Coffea arabica* L.) pulp with gelling properties. Carbohydr. Polym..

[30] Luo Q., Zhang J.R., Li H.B., Wu D.T., Geng F., Corke H., Wei X.L., Gan R.Y. (2020). Green extraction of antioxidant polyphenols from green tea (*Camellia sinensis*). Antioxidants.

[31] Levigne S., Thomas M., Ralet M.C., Quemener B., Thibault J.F. (2002). Determination of the degree of methylation and acetylatioin of pectins using a C18 column and internal standards. Food Hydrocoll..

[32] Cangussu L.B., Melo J.C., Franca A.S., Oliveira L.S. (2021). Chemical characterization of coffee husks, a by-product of *Coffea arabica* production. Foods.

[33] Lang R., Yagar E.F., Eggers R., Hofmann T. (2008). Quantitative investigation of trigonelline, nicotinic acid, and nicotinamide in foods, urine, and plasma by means of LC-MS/MS and stable isotope dilution analysis. J. Agric. Food Chem..

[34] Wang L., Halquist M.S., Sweet D.H. (2013). Simultaneous determination of gallic acid and gentisic acid in organic anion transporter expressing cells by liquid chromatography-tandem mass spectrometry. J. Chromatogr. B.

[35] Wang X., Yan K., Ma X., Li W., Chu Y., Guo J., Li S., Zhou S., Zhu Y., Liu C. (2016). Simultaneous determination and pharmacokinetic study of protocatechuic acid aldehyde and its major active metabolite protocatechuic acid in rat plasma by liquid chromatography-tandem mass spectrometry. J. Chromatogr. Sci..

[36] Rolin C., Whistler R.L., Bemiller J.N. (1993). Pectin. Industrial Gums: Polysaccharides and Their Derivatives.

[37] Vriesmann L.C., de Oliveira P. (2009). Polysaccharides from the pulp of cupuassu (*Theobroma grandiflorum*): Structural characterization of a pectic fraction. Carbohydr. Polym..

[38] Voragen A.G.J., Pilnik W., Thibault J.F., Axelos M.A.V., Renard C.M.G.C., Stephen A.M. (1995). Pectins. Food Polysaccharides and Their Applications.

[39] Yuan Q., Lin S., Fu Y., Nie X.R., Liu W., Su Y., Han Q.H., Zhao L., Zhang Q., Lin D.R. (2019). Effects of extraction methods on the physicochemical characteristics and biological activities of polysaccharides from okra (*Abelmoschus esculentus*). Int. J. Biol. Macromol..

[40] Clifford M.N., Johnston K.L., Knight S., Kuhnert N. (2003). Hierarchical scheme for LC-MS^n^ identification of chlorogenic acids. J. Agric. Food Chem..

[41] Clifford M.N., Knight S., Surucu B., Kuhnert N. (2006). Characterization by LC-MS^n^ of four new classes of chlorogenic acids in green coffee beans: Dimethoxycinnamoylquinic acids, diferuloylquinic acids, caffeoyl-dimethoxycinnamoylquinic acids, and feruloyl-dimethoxycinnamoylquinic acids. J. Agric. Food Chem..

[42] Jaiswal R., Kuhnert N. (2010). Hierarchical scheme for liquid chromatography/multi-stage spectrometric identification of 3,4,5-triacylchlorogenic acids in green Robusta coffee beans. Rapid Commun. Mass Spectrom..

[43] Jaiswal R., Kuhnert N. (2011). Identificatino and characterization of five new classes of chlorogenic acids in burdock (*Arctium lappa* L.) by liquid chromatography/tandem mass spectrometry. Food Funct..

[44] Veitch N.C., Grayer R.J. (2008). Flavonoids and their glycosides, including anthocyanins. Nat. Prod. Rep..

[45] Nazemiyeh H., Bahadori F., Delazar A., Ay M., Topçu G., Nahar L., Majinda R.R.T., Sarker S.D. (2008). Antioxidant phenolic compounds from the leaves of *Erica Arborea* (Ericaceae). Nat. Prod. Res..

[46] Faria A.F., Marques M.C., Mercadante A.Z. (2011). Identification of bioactive compounds from jambolão (*Syzygium cumini*) and antioxidant capacity evaluation in different pH conditions. Food Chem..

[47] Heeger A., Kosińska-Cagnazzo A., Cantergiani E., Andlauer W. (2017). Bioactives of coffee pulp and its utilization for production of cascara beverage. Food Chem..

[48] Nzekoue F.K., Angeloni S., Navarini L., Angeloni C., Freschi M., Hrelia S., Vitali L.A., Sagratini G., Vittori S., Caprioli G. (2020). Coffee silverskin extracts: Quantification of 30 bioactive compounds by a new HPLC-MS/MS method and evaluation of their antioxidant and antibacterial activities. Food Res. Int..

[49] Morimura K.I., Gatayama A., Tsukimata R., Matsunami K., Otsuka H., Hirata E., Shinzato T., Aramoto M., Takeda Y. (2006). 5-O-Glucosyldihydroflavones from the leaves of *Helicia cochinchinensis*. Phytochemistry.

[50] Jiang C.L., Tsai S.F., Lee S.S. (2015). Flavonoids from *Curcuma longa* leaves and their NMR assignments. Nat. Prod. Commun..

[51] Abdel Salam N.A., Ghazy N.M., Shawky E., Sallam S.M., Shenouda M.L. (2018). Validated HPTLC method for dihydrokaempferol-4′-O-glucopyranoside quantitative determination in *Alcea* species. J. Chromatogr. Sci..

[52] Ning Z.X., Zhan Y., Gao J.H. (2005). Synthesis of ampelopsin-7-O-α-D-glucopyranoside and its curative effect to diabetes (In Chinese). J. Food Sci. Biotechnol..

